# A Novel Metaphor Graph Drawing Method for Multidimensional Data Visualisation and Its Case Study on COVID-19 Vaccination Analysis

**DOI:** 10.3390/ijerph192315547

**Published:** 2022-11-23

**Authors:** Xin Chi, Jie Hua, Xiao Ren

**Affiliations:** Faculty of Information Engineering, Shaoyang University, Shaoyang 422000, China

**Keywords:** COVID-19, graphical drawing, metaphor, multidimensional data, Nightingale rose chart, visualisation

## Abstract

Visualisation techniques have been one of the best data processing and analysis methods in recent decades, and they have assisted in data understanding efforts in various fields. Visualisation techniques for low-dimensional data are well developed and applied in multiple sectors; however, multidimensional data visualisation techniques still present some limitations, such as inaccurate data comparison and perception, exaggerated visual differences, label occlusion, and overlap. This study addresses the pros and cons and proposes a novel graphical drawing method, the multidimensional rose chart. It adopts the design idea of the Nightingale rose chart, but overcomes relevant limitations. The main challenges of this area include the incomplete presentation of multidimensional data, the neglect of the linkage of multiple attributes, the inefficient use of space, and the lack of simplicity of the interface. Contributions include enriching the representations of multidimensional data through the use of colour shades, area, and height sizes to represent values; straightforward data attribute comparisons via graph nesting; and detailed attributes showing the use of specific value labels. To verify the preliminary validity of this method, we imported COVID-19 data into experiments and further compared the final layouts with traditional methods, such as the line chart, bar chart, tree, parallel coordinate chart, and Nightingale rose chart, as well as their structures, functionalities, clear advantages, and disadvantages. The experimental results show that multidimensional rose diagrams perform effectively in presenting multidimensional data when comparing other graph drawing methods in our case, and the outcomes match existing works’ conclusions in related COVID-19 research sectors. This work has the potential to provide a suitable supplemental approach to the multidimensional data analysis.

## 1. Introduction

With the advancements in time and technology, information data are increasing at an alarming rate and complexity [1]. This has led to a massive challenge in the processing and management of complex datasets, with one of the convenient ways in which this challenge can be addressed being through data visualisation [2]. When processing information data, two aspects are generally included, namely, the dimensionality of the data and the visualisation of the data. In terms of the dimensionality of the data, for presenting low-dimensional data (one-dimensional data, two-dimensional data, and three-dimensional data), it is possible to find easy-to-understand explicit metaphors and models from the surrounding reality. However, when presenting multidimensional data, the complexity of their dimensions leads to this approach not being applicable [3]. The primary approach in dealing with multidimensional data is to convert high-dimensional data into low-dimensional data for processing [4]. In terms of the visualisation related to this topic, the main aspects focus on the visualisation of multidimensional/non-multidimensional data. In this paper, we focus on the processing and analysis of multidimensional data from a visualisation perspective.

In visualising non-multidimensional data, statistical graphics can convey information about the data [5]. Traditional graph drawing methods, such as line charts [6], bar charts [7], pie charts [8], and trees, are often applied to visualise two-dimensional datasets on data trends, hierarchy, and other aspects [9,10]. They require little knowledge to understand. However, they cannot be adopted to present multidimensional data.

The visualisation of multidimensional data mainly comprises the presentation of different and identical views of the same dataset through visualisation techniques [11]. Commonly used statistical graphs include scatter charts, parallel coordinate charts, and Nightingale rose charts. There are several major scenarios where these methods have practical use. In a cartesian coordinate system, scatter charts are often used to view trends in data and identify outliers, determine if there is a correlation between the variables, and find stepwise or the aggregation of data. It has the advantage of showing the distribution and aggregation of data, and the disadvantage of being visually confusing, with essentially only correlations, distributions, and aggregations visible [6]. Parallel coordinate charts are typically used to graph individual data elements spanning multiple dimensions; each dimension is associated with a vertical axis, and each data object is shown as a series of connected points along the dimension/axis. It has the advantage of being able to handle multidimensional data and the disadvantage of the data being dense, due to which the graph tends to become cluttered and the lines illegible [6,12]. In a polar coordinate system, Nightingale rose charts are often used to represent the magnitude of each value about the total, the change in the data over some time, and the cyclical time pattern. It has pros, such as highlighting differences in sizes between different categories, explaining the cyclical nature of seasonal/circular data very well, and having a high space utilisation. The cons include the judging area and angle not being as accurate as perceiving the spatial length and position, not being conducive to data comparisons, it can visually exaggerate the differences between data (e.g., the area difference between data can be 1%, but the visual difference is more than 1%), and when the number of sectors is large, and some of the sector values are small, the problem of overlapping labels and mutual occlusions can easily occur [13,14,15]. The methods above can be applied to representing multidimensional data practically, but they also come with some limitations.

This study proposes a novel graphical drawing method to address the limitations of visualising statistical graphics for multidimensional data. The method is based on the Nightingale rose chart with improvements to aspects such as retaining its advantages of highlighting differences, interpreting periodic features, and high spatial utilisation, and addressing the disadvantages of inaccurate data comparison and perception, exaggerating visual differences, and overlapping multidimensional data labels. Additionally, some interaction features are added to assist users in better understanding complex data. This study also provides preliminary evidence for the advantages of the novel graphical plotting method in terms of its high spatial utilisation, the high dimensionality of applicable data, simplicity of the interface, and accuracy of data perception and comparison through an epidemic (COVID-19) data-related use case.

The rest of this article is divided into several sections. Section 2 presents the methodology and specific design of the novel graphic drawing method. Section 3 provides examples of using novel/traditional graphical plotting methods on epidemic datasets. In Section 4, a comparison of the advantages and disadvantages of novel and traditional methods of drawing graphics is given, as well as an overview of the final layout. Future work and conclusion are provided in Section 5.

## 2. Methodology

This section introduces two new methods of drawing metaphorical graphs—the multidimensional rose diagram and the stacked multidimensional rose diagram. Both methods were primarily used to present multivariate/multidimensional data and their complex multivariate relationships. In contrast, visual metaphors can be used as a natural way to map categorical variables to visible entities, conducting effective interactive exploration, and performing analyses [16]. Therefore, these two new methods are supported by the multidimensionality and comprehensiveness of the data they cover. They can also potentially be used to assist in the effective analysis of data through user interactions. Apache ECharts was used for the implementation [17]. The interaction was still in progress at this stage. Features that were initially implemented included a hover display, data filtering/sorting, and component communication. In detail, the petal was highlighted, and relevant information was shown in the stamen section by hovering over the petal. Petals could be filtered and sorted by specific attributes that the petal clicked. The rose graph could exchange data with other components, such as a table, parallel coordinates, etc., which could enrich the dashboard’s capability of analysing data from a more comprehensive perspective. More features are planned in future works.

Both graph drawing methods were based on the Nightingale rose diagram (Florence Nightingale). This rose diagram, also known as a polar zone diagram, is a circular histogram, originally used to express seasonal death rate data from military hospitals [18]. Its structure is very similar to that of a rose and can be used to display more information. It uses circles and lends itself to a time-based genre to show the progression of data. It is plotted from the centre of the circle, with the angle from the centre remaining the same for each segment, and the square root of the radius varying with the data. The value obtained by dividing each data value by the sum of the data is the size of the slice [19], i.e., the size of the data. Each area can also be coloured according to the data attributes represented and used to represent a different cause of death. In simple terms, the Nightingale rose is a polarised bar chart that transforms the bar into a more aesthetically pleasing pie chart format. Unlike pie charts, which use angles to represent values or percentages, Nightingale rose charts use the sector’s radius to indicate the data’s size, the colour to indicate the type of attribute, and the angle of each sector’s sector is kept consistent.

**Figure 1:**
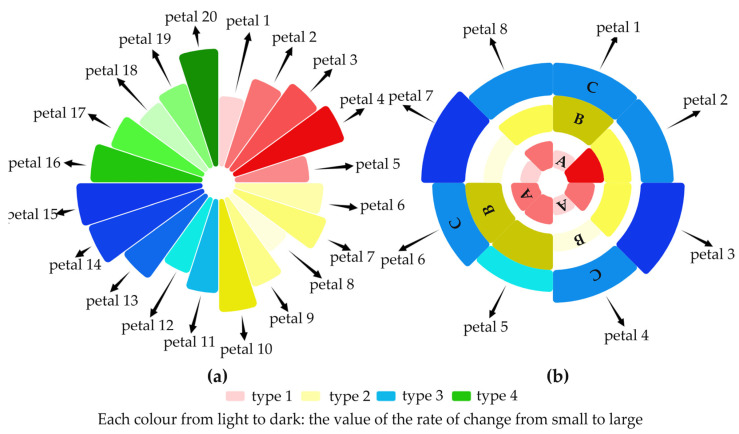
Examples of the proposed method. (**a**) Multidimensional rose chart. (**b**) Stacked multidimensional rose chart.

The traditional Nightingale rose chart has a single attribute and category, which has some limitations (inaccurate data comparisons and perceptions, exaggerated visual differences, overlapping and obscured labels when too much data are available, etc.) when handling multidimensional data [13,14,15]. Novel methods have kept similar layouts to the Nightingale rose diagram, but use different representations of multidimensional data, showing, for example, layers of petals, shades, and types of colour, etc., to represent complex data to address the limitations accordingly. Examples of the two graph layout methods can be found in Figure 1, and the graph hierarchy details can be seen below.

Figure 1a shows a multidimensional rose diagram with 20 petals, in which multiple objects that come with the same data attribute sets can be represented, or they can be used to present multiple data attributes of an object. The size of the petal area can indicate the value, time, attribute type, etc. For example, through their size, petal one shows the smallest value, and petal four shows the largest one. The value here could be used to reveal the COVID-19 cases, population size, or date; basically, any numerical values. The petals’ type and shade of colour can also indicate relevant attributes and values. For example, four colours in Figure 1 represent four types, whereas petals one to five are red, demonstrating one data attribute. The lighter to darker colours can symbolise smaller to larger values and indicate that the categories are ordered from front to back. This is reflected in the fact that the darker the red colour, the larger the value. Each petal was also divided equally from the centre of the graph in this case.

Figure 1b offers a stacked multidimensional rose diagram, which can be viewed as being nested from multiple petals. The innermost petals one to eight are similar to those in Figure 1a, representing relevant values. However, each petal was subdivided into several levels, such as A, B, and C, to show data attributes. The structure is similar to a stacked bar chart, and can be used to signify additional features. The order of A, B, and C can also be customised. Similarly, the size of the petals can indicate relevant values, and the petal’s type and shade of colour can also represent relevant attributes and values. Three primary colours were used in this example to describe the three attributes, with the lighter to darker colours representing smaller to larger values.

The two graphs in Figure 1 are mainly employed for data comparison and analysis purposes, and the method can be applied to a wide range of scenarios, especially for multidimensional data analyses, such as epidemic, air quality, finance, education, and healthcare data analytics. In general, the proposed graph drawing method provides favourable conditions for analysing multidimensional data in a user-friendly manner. The use case provided in Section 3 is for the visual analysis of vaccines’ impacts on epidemics. Two years of data were collected on four changing rates from six countries during the epidemic and multidimensional rose plots were generated. Relevant data were further compared for the same country (e.g., comparing vaccination rates with death rates and infection rates in the USA) and different countries (e.g., comparing vaccination rates in the USA and India). Patterns could be observed by comparing adjacent, same-level, and different-level petals. Compared to traditional graph methods, this method exhibited a relatively high spatial utilisation, the data presentation was more comprehensive, the graphical interface was simpler, and the comparison and perception of countries and rates of change were more accurate. The use case further revealed that the decrease in death rates during the epidemic was the effect of receiving an increase in vaccination rates in most countries. However, a rigorous analysis is needed to support the outcomes, which are planned to be included in future works. The present format at this stage was only used as additional evidence to relevant existing research.

## 3. Use Case

The use cases of two proposed methods were given based on a dataset related to COVID-19, including information on the sample dataset, the process of transforming the data into a multidimensional rose diagram and drawing the surface, a description of the relevant cases, and a comparison with traditional visual statistical graphics.

The sample dataset included four rates of change (death rates, infection rates, vaccination rates, and complete vaccination rates) from six countries (the USA, India, Brazil, the UK, France, and Russia) during the epidemic (January 2020–January 2022). These data were essentially downloaded and collected from the WHO [20]. The death rate is the value of the cumulative number of deaths divided by the cumulative number of infections in the six countries mentioned above during the epidemic [21]. The infection rate is the cumulative number of infections divided by the cumulative number of nucleic acid tests in the six countries mentioned above during the outbreak [22]. Similarly, the vaccination rate is the value of the cumulative number of people in six countries who received at least one vaccine dose during the epidemic divided by the total number of people in that country [23]. The complete vaccination rate is similar to the vaccination rate, and it is the value of the cumulative number of people who received all vaccine doses divided by the total number of people in the country [23].

**Figure 2:**
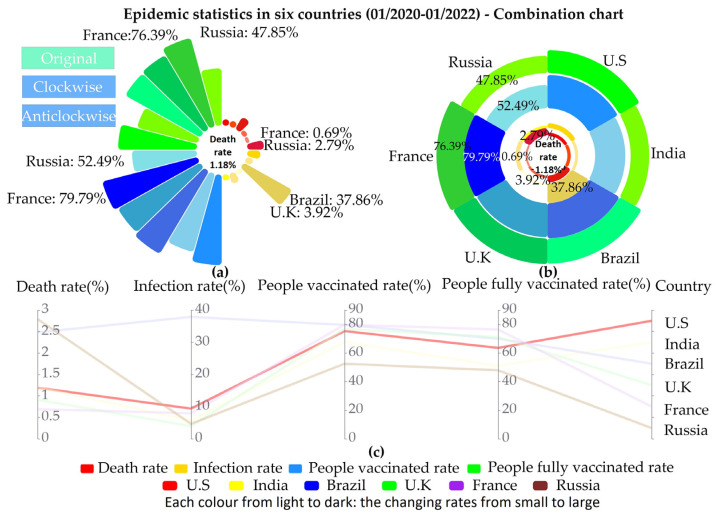
Epidemic statistics in six countries (January 2020–January 2022). (**a**) Multidimensional rose chart. (**b**) Stacked multidimensional rose chart. (**c**) Parallel coordinate chart.

The process of transforming the data into a multidimensional rose diagram and plotting the surface was as follows: In the first step, the downloaded and collected data were processed and formatted according to the definitions of the four rates of change mentioned above to obtain the final data, the details of which can be found in Table 1. In the second step, the data were transformed into a graph. This was divided into two parts, a and b, in Figure 2. Since a multidimensional rose plot is similar to a bar chart or an equal pie chart in polar coordinates, each petal was assigned equally. Therefore, the process of implementing part a was as follows: As there were four rates of change in the data, the petals were divided, overall, into four sections, each with a 90 degrees angle of roundness. Each part was further divided into six petals, each with a circular centre angle of 15 degrees. The value of each petal was expressed in terms of the radius and area, which was then taken as the value of each rate of change for each country (i.e., the values in Table 1). If the values were too large to display on the screen, an appropriate size could be chosen in proportion to each value. For the implementation of part b, the process was similar to a. The difference was that, here, there were only six petals, representing six countries, and each petal had a circular angle of 60 degrees. Each petal had four layers, and each layer represented a rate of change, the size of which was expressed in the radius or area, the value of which was the value of the specific rate of change. In the third step, colour coding was performed. The final data were sorted, and four base colours were assigned to the four rates of change. The shade of colour for each rate of change depended on the size of its value; the smaller the value, the lighter the colour assigned. In the fourth step, we followed the above method of programming using the JavaScript language. In the fifth step, the details were optimised and the final chart was obtained.

**Figure 3:**
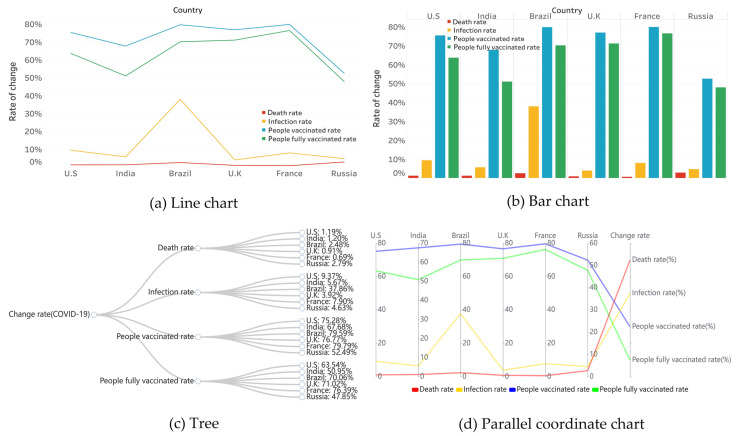
Epidemic statistics in six countries (January 2020–January 2022)-traditional chart. (**a**) line chart. (**b**) bar chart. (**c**) tree. (**d**) parallel coordinate chart.

This section presented concrete examples of two novel graph drawing methods and traditional graphic drawing methods. Figure 2 shows concrete examples of the two new graphical drawing methods and Figure 3 displays concrete examples of the traditional graph drawing method.

### 3.1. Use Case of the Proposed Methods

Figure 2 represents a concrete example of the new graphic drawing method, where a is a multidimensional rose plot, b is a stacked multidimensional rose plot, and c is a parallel coordinate plot.

In Figure 2a, the multidimensional rose diagram, twenty-four petals were divided into four sections using four primary colours, representing four changing rates in six countries, in which red corresponds to the death rates, yellow to the infection rates, blue to the vaccination rates and green to the complete vaccination rates. Additionally, each section consisted of six petals corresponding, in clockwise order, to six countries, namely, the USA, India, Brazil, the UK, France, and Russia. The size and length of the area in each petal represent the magnitude of the rate of change value. The shade of each colour also represents the range of magnitude of the change rate values, as shown by the fact that the darker the colour, the larger the value. For example, the maximum value of the vaccination rates in the graph was for France, and the minimum value was for Russia. The breakdown was as follows: in the vaccination rates section (blue section), the area representing the French petals was the largest and the darkest; the area representing the Russian petals was the smallest and the lightest. The graph also marked the maximum/smallest value for each rate of change. For example, the maximum value for the death rate in Russia was 2.79%, and the minimum value for the death rate in France was 0.69%. Values for other countries’ rates of change could be viewed via the interactive function (hovering over the petal one wished to view would display specific data information in the stamen section). For example, when we selected the death rate petal representing the US, the pistil would show the death rate and the specific value. Additionally, to avoid excessive differences in the size of the values, resulting in the visual presentation of petal sections with small values being too small to observe and compare, we scaled the values by a factor of 5 and 1.2 for each country’s death and infection rates, respectively.

In Figure 2b, the stacked multidimensional rose diagram, six petals represented six countries in clockwise order. Each petal was divided into four layers, meaning, from the inside out, death rates, infection rates, vaccination rates, and complete vaccination rates were indicated in red, yellow, blue, and green, respectively. The size and length of the petal area and the shade of each colour represented the magnitude of the rate of change value. The larger the area, the greater the changing value; the darker the colour, the greater the value of changing. For example, the graph showed that the maximum value for the complete vaccination rate was in France, and the minimum value was in Russia. The breakdown was as follows: in the complete vaccination rates section (green section), the area represented with the French petals was the largest and the darkest; the area represented with the Russian petals was the smallest and the lightest. The maximum/smallest values for each rate of change were annotated in the graph. For example, the maximum vaccination rate was in France (79.79%) and the minimum was in Russia (52.49%). Information on data on rates of change for other countries could be viewed through the interactive hover display function. For example, if we selected the petal representing the US death rate, the stamen section would show the death rates and specific values.

Looking at the concrete examples above, some facts were discernible. In Figure 2a, the darker the green and blue petals were, the larger their areas; the lighter the corresponding red petals (same countries) were, the smaller their areas. This revealed that the higher the vaccination rate and complete vaccination rate, the lower the mortality rate. This could be explicitly seen in the example of France, where, for example, the vaccination rate (79.79%) and the complete vaccination rate (76.39%) were the highest, and the death rate (0.69%) was the smallest. Russia showed the opposite (the lowest vaccination and complete vaccination rates came with the highest mortality rates), which could also shed light on the effect of vaccination rates on mortality. However, this would require the inclusion of other potential factors not included in the study. The findings could also be applied to the petals representing the USA, India, and the UK. On the other hand, the shades and area sizes of the green and blue petals did not have a significant effect on the shades and area sizes of the yellow petals, suggesting that the effect of vaccination rates on infection rates was not visually obvious based on this graph drawing method, and that the next stage would be to include more data over time and improve the proposed method to explore the relationship in depth. In Figure 2b, similar results could be found. That is, the green and blue parts of the interior of each petal were darker and larger in area, while the red parts of the interior of that petal were lighter in colour and smaller in size. However, in Figure 2, part b was compared in a more intuitive way than part a. For example, in France, the vaccination rates, complete vaccination rates, and death rates were at different levels of the same petal, making it easier to compare and analyse. Again, these findings could only be applied to the USA, India, the UK, and France. Additionally, within each petal, the darker and larger the green and blue parts of the outer petals, the darker and larger the yellow parts of the inner petals, which suggested that the greater the vaccination rates and complete vaccination rates, the greater the infection rates. These facts could also be seen in the USA, Brazil, and France. For example, Brazil had relatively high vaccination rates (79.59%) and complete vaccination rates (70.06%), while having the highest infection rates (37.86%). India, the UK, and Russia, on the other hand, showed the opposite situation. Again, this suggested that vaccination rates had no intuitive effect on infection rates. In conclusion, without considering other facts, the decrease in death rates in most countries was influenced by the increase in vaccination rates, and vaccination had no direct effect on the change in infection rates. These findings were consistent with some of the conclusions of existing studies [24,25,26].

In addition, some findings could be observed in Figure 2. Firstly, the multidimensional rose chart was more spatially efficient and had a more straightforward interface due to the adoption of fewer information labels and reasonable component space arrangements. Secondly, it was suitable for presenting high-dimensional data, as it could simultaneously show data such as the country, rate of change, value size, colour type, shade, and time. Thirdly, multiple data attributes could be compared horizontally, vertically, clockwise, and counterclockwise, and presentations could be processed through the area size, height, and colour shade. Hence, the multidimensional rose charts were relatively comprehensive in terms of data perception and comparison.

In Figure 2c, the parallel coordinate chart, six colours represented the six countries, and the four axes represented the four rates of change. Some interaction features were added, and more functions are in the progress of being planned. At this stage, on the dashboard in Figure 2a, b and c were linked. Clicking on any petal in a or b would reflect the relevant data representation on other graphs through fading and filtering, as well as detailed data of the clicked petal showing in the stamen (centre) section in a and b. On the other hand, the red line representing the USA was highlighted accordingly in c, with the other lines (countries) fading. In addition, the petals in the multidimensional/stacked multidimensional rose diagram could be switched clockwise/anticlockwise using attributes when the corresponding button in the upper left corner of the chart was clicked. For example, when we clicked on the clockwise button, the petals in each rate of change section in part a were sorted clockwise by size. Compared to the previous method of sorting by country only (original order), such sorting (clockwise/counterclockwise order) allowed for better data information acquisition and comparison. For example, when sorting clockwise, we could compare the maximum/smallest value of each rate of change for each country and obtain some helpful information from it. Of course, other data information and some conclusions could also be mined and analysed from the combination of these three sorting types. The dashboard, currently only in its initial stage, combined the advantages of the stacked multidimensional/stacked multidimensional rose charts and parallel coordinate charts to provide the user with an overview and detailed data, therefore, providing a more comprehensive visual result for analysis.

### 3.2. Use Cases of Traditional Methods

Figure 3 represents specific examples of the four traditional graphing methods, where a and b applied traditional line and bar charts showing the rate of change in six countries. They required little expertise to observe relevant data in the present format, such as the ability to see trends based on periods and to compare the performance of an attribute across countries horizontally, but only exceled at datasets with limited attributes. Figure 3c shows a tree that focuses on the hierarchical relationship between the six countries and the four rates of change, but did not allow for the viewing of the relevant data’s details. Figure 3d shows a parallel coordinate chart, which offered multiple data attributes and their relationships, and the disadvantage of inaccurate data comparison and perception, as well as arbitrary label overlap problems when the data volume was large, etc.

## 4. Discussion

This section introduces the similarities, differences, advantages, and disadvantages of line, bar, tree, parallel coordinate [27], Nightingale rose, and multidimensional rose diagrams. The details and functionality of the final layout (the dashboard of the case) and possible future visual interaction features are also presented.

Based on the above description of the case diagrams of the two new graph drawing methods and the traditional graph drawing method, we could find some similarities and differences. In terms of similarities, the three thematic objects of the cases could be seen in all the graphs, namely, the epidemic, the six countries, and the four rates of change. In terms of differences: (1) the size of the blank parts was different. In the case of presenting the same data information, the graph of the new graph drawing method had fewer empty parts than the graph of the traditional drawing method. (2) The level of dimensionality was different. The new graph used the area size, colour type, and colour shade to describe the relationship, while the traditional chart only took area sizes and colour types to describe the relationship. (3) The level of detail of the data was different. Compared with the new kind of diagram, the data information of the traditional diagram was relatively more detailed. (4) The coordinate systems were different. The new chart used a polar coordinate system, while the conventional diagram used a right-angle coordinate system and a parallel coordinate system.

In general, the diagrams of the two new graph drawing methods had advantages and disadvantages compared to the charts of the traditional graph drawing methods. In terms of benefits, the novel approach addressed some issues of existing works: it came with better space utilisation, in that the graph was more compact and allowed for more information to be displayed in a limited space; it was also capable of representing higher dimensions of data compared to traditional graph drawing methods; and the interface was more straightforward; moreover, the present interaction features enabled the initial comparisons among attributes and, hence, allowed for it to be possible to discover potential connections in between. In terms of the disadvantages, this proposed method required some expertise or training, and it was relatively more difficult to understand than traditional graphs; the interaction features at this stage were relatively simple and are planned to be improved in future works.

**Figure 4:**
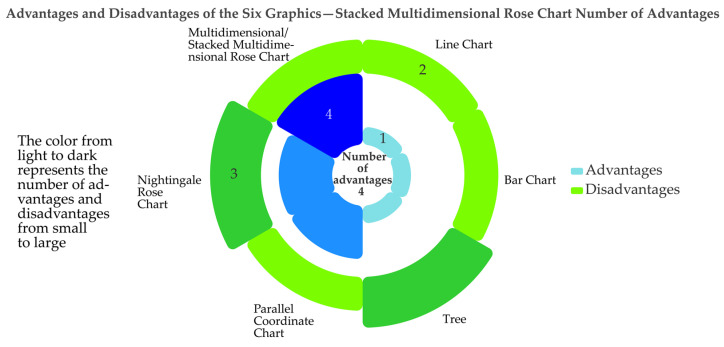
Advantages and disadvantages of the six graphics—stacked multidimensional rose chart. (‘1’, ‘2’, ‘3’, and ‘4’ here indicates how many advantages the relevant graph method has, and details are in Table 2.)

Below is an overview of the six statistical graphics mentioned above and a description of their advantages and disadvantages. The specific advantages and disadvantages can be found in Table 2 and Figure 4.

Firstly, an overview of the six graphs mentioned above and their advantages and disadvantages can be viewed in Section 1 and were not presented here. Figure 4 shows a stacked multidimensional rose diagram’s six graphical advantages and disadvantages. These included the following features: (1) different petals representing different graphs in a clockwise order, a line chart, bar chart, tree, parallel coordinate chart, Nightingale rose chart, and multidimensional rose chart; (2) the type of petal colour representing the advantages and disadvantages (for example, blue represented advantages and green represented disadvantages); (3) petal colour shades, area sizes, and height sizes all indicated the number of strengths and weaknesses. The colour from light to dark indicated the number of small to large Covid cases. The larger the area and height, the greater the number of advantages and disadvantages. For example, the petals representing the bar graph had the lightest colour, the smallest area and height, and the smallest number of advantages (the number of advantages was one). Petals representing other graphs were expressed in the same way. Additionally, when we selected a petal representing a multidimensional rose graph, the pistil part would show four, indicating that the multidimensional/stacked multidimensional rose graph had four advantages.

Secondly, it was described how the novel graphic drawing methods were an improvement in response to the shortcomings of the Nightingale rose chart. This paper’s novel graphic drawing method inherited the advantages of the Nightingale rose chart, while making some improvements to address its drawbacks. Firstly, for perceived inaccuracies, we added colour coding, i.e., the use of colour shades to indicate the size of the value. Secondly, for the data comparison, we used multiple nested rose charts to facilitate a comparison. That is, values for the same attributes were placed on different levels of the same petal or on other petals of the same rose diagram. Thirdly, in terms of visual differences, we used the area size and height to indicate values to prevent exaggerated differences. That is, the values were not only expressed in terms of area, but also in terms of height. Fourthly, for label overlaps and masking, we used the method of showing partial labels. This simply meant that only representative values (maximum/minimum, specific values, etc.) were displayed in tabs, and other values could be viewed by selecting them with the mouse.

**Figure 5:**
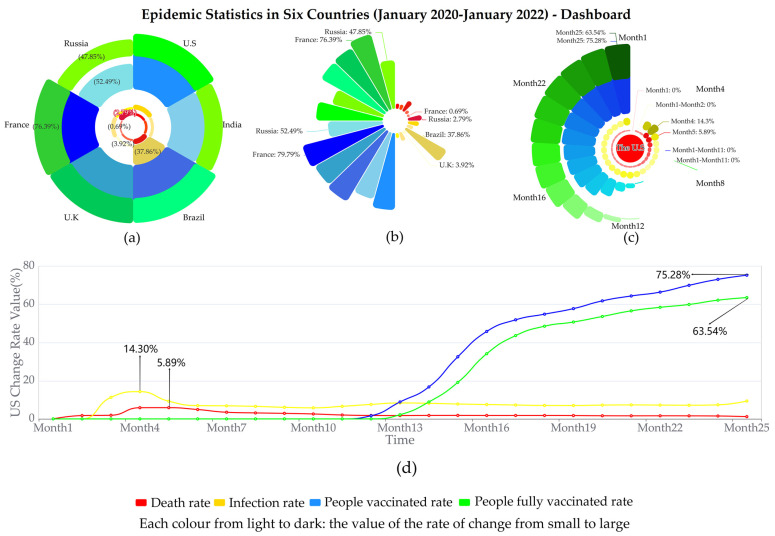
Epidemic statistics in six countries (January 2020–January 2022)-dashboard. (**a**) Stacked multidimensional rose chart. (**b**) Multidimensional rose chart. (**c**) USA data-combined stacked multidimensional and multidimensional rose charts. (**d**) USA data-line chart.

Finally, the details and functions of the case dashboard are presented. The dashboard was the final layout of the multidimensional/stacked multidimensional rose chart. In terms of detail, a and b in Figure 5 were the same as b and a in Figure 2, which we already covered in Section 3 and did not further explain here. c represents information on the four rates of change in the United States over 25 months, which combined the characteristics of a and b. The colour of the petals represented the type of the rate of change, the length and colour shade of the petals indicated the size of the value, the labels showed the maximum/minimum value for each rate of change, and the stamens indicated the country’s information. In a clockwise direction, the petals represented the 25 months in turn, with each petal divided into four levels (four rates of change). d is a line graph with the same data content as c. The horizontal axis represented the time, the vertical axis represented the value, the colour represented the rate of change, the height of the points represented the size of the rate of change value, and the lines indicated the trend of the rate of change over time. Additionally, marked on the graph were the maximum values for each rate of change. In terms of functionality, to facilitate data comparison, the four sections of a, b, c, and d used the same colour to indicate the type of rate of change and the same shade of colour to indicate the magnitude of the value. A comparison of the different rates of change for the same countries in the four sections, as well as aspects of the same rates of change for other countries, revealed some relationships between the countries and the various rates of change under the epidemic.

In summary, the use case in this study imported COVID-19-related data and applied the proposed method to generate multidimensional rose graphs. What was concluded was that, in most cases, the decline in death rates in most countries was influenced by an increase in vaccination rates, realized through comparing petal sizes and the colours at the adjacent, same, and different levels. The result matched other related existing studies, and offered more evidence from a visual analysis perspective. Additionally, the use case also demonstrated the advantages of multidimensional rose diagrams in terms of high spatial utilisation, interface simplicity, suitability for high-dimensional data presentation, and the accuracy of data comparison and perception. At this stage, the present research focused on multidimensional data visualisation and its application scenarios in COVID-19 data analytics.

## 5. Conclusions and Future Works

This study presented a multidimensional rose diagram, a novel graph drawing method for a better presentation of multidimensional data. This graph drawing method was built based on the structure and features of the Nightingale rose chart, retaining its strengths and addressing some of its limitations. Compared to other statistical graphics, the novel graphing method had the advantages of high space utilisation, higher dimensionality of the data displayed, simple user interface, accurate data perception, and easy comparison. However, there were still limitations, such as the relative difficulty of understanding the graphics and the limited interaction feature at this stage. More knowledge may be needed in a specific area to obtain insights into complex datasets rather than traditional methods. In addition, we used some use cases to illustrate how multidimensional rose diagrams can assist readers in quickly analysing and processing data information in multidimensional datasets.

At this stage, the present research focused on multidimensional data visualisation and its application scenarios in COVID-19 data analytics and only demonstrated the initial effectiveness of the graph method. There is still a need for improvements in graph understanding and user interaction, etc.; thus, we plan to include the following aspects in future works: In terms of targeting the relative difficulty of experience graphics, more detailed instructions should be provided, which consist of hint descriptions, graphic splits, and demonstrations of manipulation. For the visual interaction, rich visual interaction features should be added, including the filtering of data, querying of data information, the transformation of graphs (bar, line, and multidimensional/stacked multidimensional rose charts), zooming in/out of areas, interface reduction, and data linkage, which means that by selecting attributes from individual components we could reflect on other components. The method’s effectiveness needs to be further assessed through the use of essential graph drawing aesthetic criteria and questionnaires [28], with which epidemiological experts can be involved in. Hence, this proposed method could not only provide more evidence to existing studies, but also bring insights into complex datasets.

## Figures and Tables

**Table 1:** Death rate, infection rate, vaccination rate and complete vaccination rate in six countries.

Country	Death Rate (%)	Infection Rate (%)	Vaccination Rate (%)	Complete Vaccination Rate (%)
USA	1.18	9.37	75.28	63.54
India	1.18	5.67	67.68	50.95
Brazil	2.48	37.86	79.59	70.06
UK	0.91	3.92	76.77	71.02
France	0.69	7.90	79.79	76.39
Russia	2.79	4.63	52.49	47.85

**Table 2:** Advantages and disadvantages of the six graphics.

Types	Advantages	Disadvantages
Line	Indicates the magnitude and trend of the value	1. Cannot display multidimensional data; 2. cannot display the percentage of the situation
Bar	A clear indication of the magnitude of the various values	1. Does not apply to larger datasets; 2. does not show percentages
Tree	Clear display of hierarchy and hierarchical data	1. Cannot display multidimensional data; 2. lack of detailed content information; 3. cannot display the percentage situation
Parallel Coordinate	1. Integrity of results; 2. connectivity of results; 3. consequences of results	1. The graphs and lines are too cluttered when there is too much data; 2. it is difficult to find the correlation analysis between all axes other than the adjacent axes
Nightingale Rose	1. Highlights differences in size across categories; 2. explains the cyclical nature of seasonal/circular data; 3. high space utilisation	1. Inaccurate data perception; 2. not conducive to data comparison and exaggerated visual differences; 3. more data can trigger label overlap and obscuration
Multidimensional Rose	1. High space utilisation; 2. can display higher dimensional data; 3. simple interface; 4. accurate data perception and easy comparison	1. Relatively difficult to comprehend; 2. interactivity is not good enough
